# A Multiloculated Mirage: Imaging Diagnosis of a Pediatric Cystic Nephroma

**DOI:** 10.7759/cureus.108124

**Published:** 2026-05-01

**Authors:** Prashant Ghogare, Abhinav Chander Bhagat, Pallem Harshavardhan, Roshan Chanchalani, Pramod K Sharma

**Affiliations:** 1 Radiodiagnosis, All India Institute of Medical Sciences, Bhopal, Bhopal, IND; 2 Pediatric Surgery, All India Institute of Medical Sciences, Bhopal, Bhopal, IND

**Keywords:** cystic nephroma, cystic renal lesion, multiloculated cystic lesion, pediatric renal tumor, renal neoplasm

## Abstract

Cystic nephroma (CN) is an uncommon benign pediatric renal neoplasm composed of multiloculated cysts separated by fibrous septa, lacking solid or blastemal elements. It occurs primarily in toddlers, often presenting as an asymptomatic abdominal mass. CN lies at the benign end of the spectrum that includes cystic partially differentiated nephroblastoma (CPDN) and Wilms tumor. We report a case of pediatric CN with detailed imaging and clinical correlation to highlight its distinguishing features.

In this report, we present a case of a one-year-old boy who presented with a three-month history of a left-sided abdominal mass. Ultrasound showed a well-circumscribed multicystic lesion at the left kidney’s lower pole, composed of anechoic loculi with thin septa (no solid component or vascularity). Contrast-enhanced CT confirmed a large (≈11 × 9 × 8 cm) encapsulated multiloculated cystic mass arising from the left kidney’s lower pole, with enhancing thin septa and herniation of cysts into the pelvicalyceal system; no enhancing nodules or calcifications were seen. The patient underwent left nephroureterectomy; grossly, the specimen showed numerous clear-fluid cysts separated by fibrous septa. Microscopically, cysts were lined by flattened-cuboidal epithelium with focal hobnail change, and fibrous septa contained only mature stroma and inflammation, with no blastema or immature elements - consistent with CN (resection margins and sampled lymph nodes were free of tumor).

## Introduction

Cystic nephroma (CN) is a rare benign renal tumor of childhood, characterized by a multicystic (honeycomb) architecture without solid tumor nodules [1,2]. Although benign, its clinical significance lies in its imaging overlap with cystic partially differentiated nephroblastoma (CPDN) and cystic Wilms tumor, both of which may require more aggressive management. Therefore, early recognition of its characteristic imaging features and radiologic-pathologic correlation is essential to guide appropriate surgical planning and avoid overtreatment [1,2]. It predominantly affects young children (typically under four years), with a strong male predominance, in contrast to adult CN, which almost exclusively occurs in middle-aged women [1]. The lesion is thought to represent the benign end of a spectrum of metanephric neoplasia that includes CPDN and Wilms tumor; indeed, CN and CPDN are histologically distinct but grossly identical, both appearing as encapsulated multilocular cystic masses. Clinically, pediatric CN usually presents as a painless abdominal mass on physical exam.

On imaging, CN is classically a sharply marginated, multiloculated cystic mass arising from the kidney, with cystic fluid and enhancing thin septa (the capsule and septa enhance). Because all solid/embryonal elements are absent, CN typically shows no enhancing nodules or coarse solid tissue [2]. However, this purely cystic appearance overlaps with CPDN and can mimic necrotic Wilms tumor or other cystic renal lesions, making radiologic differentiation challenging [1]. Therefore, definitive diagnosis requires histopathological evaluation. Notably, recent studies have documented frequent germline DICER1 mutations in pediatric CN (part of the DICER1-PPB syndrome spectrum), although genetic analysis was not performed for this patient.

## Case presentation

A one-year-old boy was evaluated for a gradually enlarging, non-tender left-sided abdominal mass noted over the preceding three months. He had no urinary tract symptoms, hematuria, or systemic complaints. Physical examination revealed a smooth, mobile, 11-13 cm mass in the left upper abdomen. Blood pressure was normal; there were no other abnormalities.

**Figure 1:**
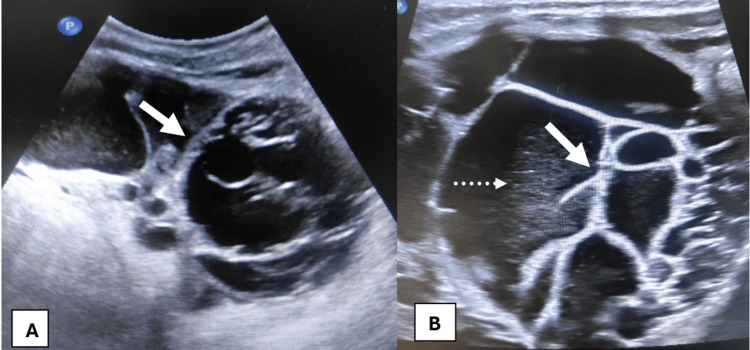
(A) Gray-scale US shows a large, multiloculated cystic lesion (arrow) at the lower pole of the left kidney. No solid component is visible. (B) US demonstrates intervening septa (arrow) and moving echoes (dotted arrow). These findings favor cystic nephroma by demonstrating a well-circumscribed, multiloculated cystic lesion without solid components, although histopathological confirmation is required. US: ultrasound

Initial ultrasound of the abdomen (Figure 1) demonstrated a well-circumscribed, multiloculated cystic mass arising from the left kidney’s lower pole. The lesion comprised numerous loculi separated by thin echogenic septa; no solid echogenic tissue or internal vascular flow was observed. This appearance is typical of CN - ultrasound is very sensitive for septa and usually shows clear fluid cysts with minimal echo and septal flow [2].

**Figure 2:**
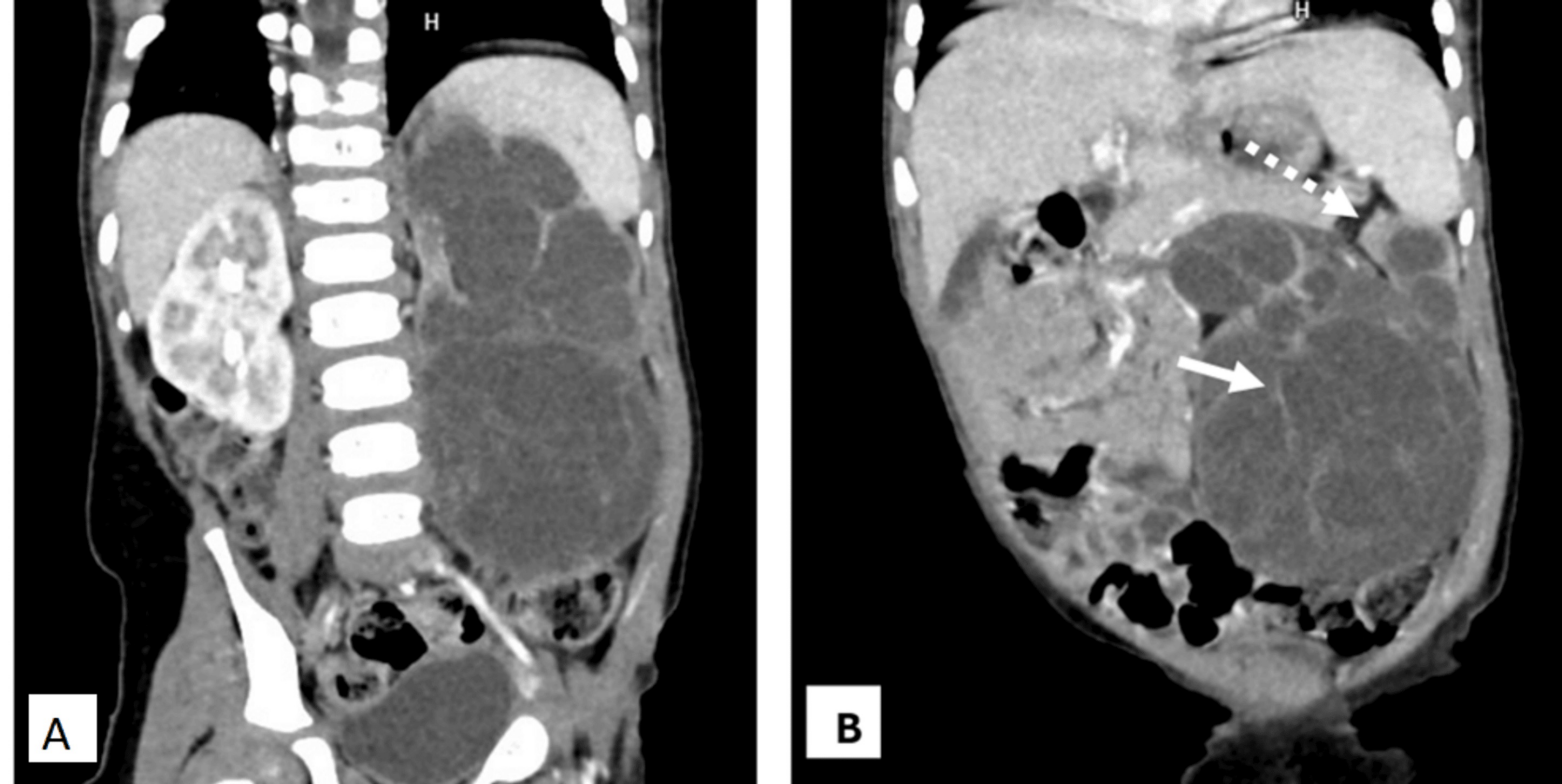
(A) and (B) Coronal contrast-enhanced images show a large, well-encapsulated, multiloculated cystic mass arising from the lower pole of the left kidney. The cystic components demonstrate fluid attenuation (mean HU ~ +10) with thin enhancing septations (white arrow). Associated hydronephrosis is noted in the upper pole, with preservation of the renal parenchyma (dotted arrows). No solid enhancing nodules or calcifications are noted. These imaging features strongly favor cystic nephroma, particularly due to the thin enhancing septa, absence of solid nodules, and herniation into the collecting system; however, overlap with CPDN necessitates histopathological confirmation. CPDN: cystic partially differentiated nephroblastoma

**Figure 3:**
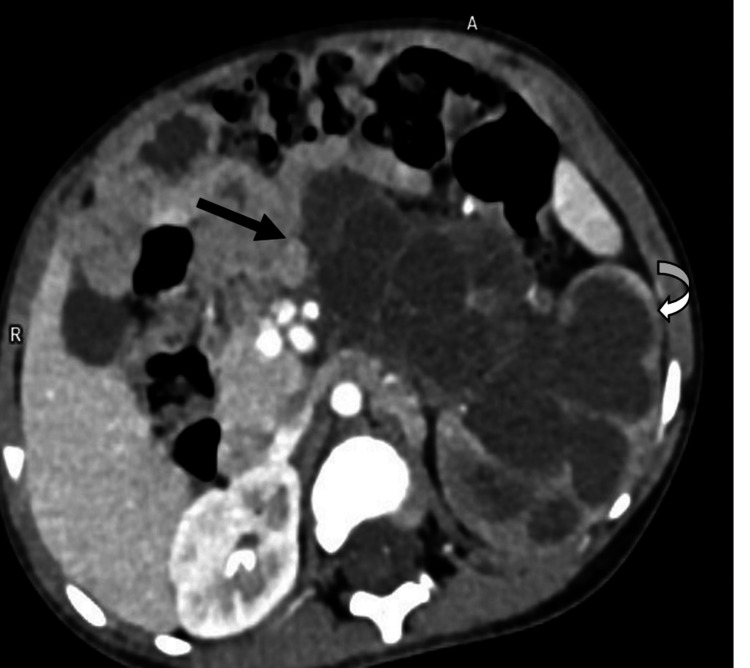
Axial contrast-enhanced image shows cystic components (black arrow) herniating into the renal collecting system, with resultant hydronephrosis and cortical thinning (curved arrow).

A contrast-enhanced CT scan was performed for further characterization. It showed a large (approximately 11.5 × 9.5 × 8.7 cm), encapsulated, multicystic mass involving the lower pole of the left kidney (Figure 2). The cystic loculi were fluid-attenuating, and thin internal septa showed mild contrast enhancement. Importantly, no solid enhancing nodules, mural thickening, or calcifications were identified. The mass displaced adjacent organs (compressing the spleen and bowels) but demonstrated no fat or vascular invasion. A characteristic finding was herniation of some cystic locules into the dilated left renal pelvis and calyces, resulting in gross hydronephrosis and cortical thinning (Figure 3) - a feature often seen in CN [2]. On delayed phases, there was no contrast excretion seen in the obstructed collecting system or left ureter. The contralateral (right) kidney was normal. No significant retroperitoneal or abdominal lymphadenopathy was identified. No evidence of distant metastasis was seen within the thorax.

Given the tumor’s size and concern for malignancy, the patient underwent a left nephroureterectomy (simple nephrectomy with ureter), with lymph node sampling via a subcostal approach. The left adrenal gland was left in situ. The operative findings confirmed complete resection of an encapsulated tumor, with no intraoperative spill or invasion of adjacent structures.

**Figure 4:**
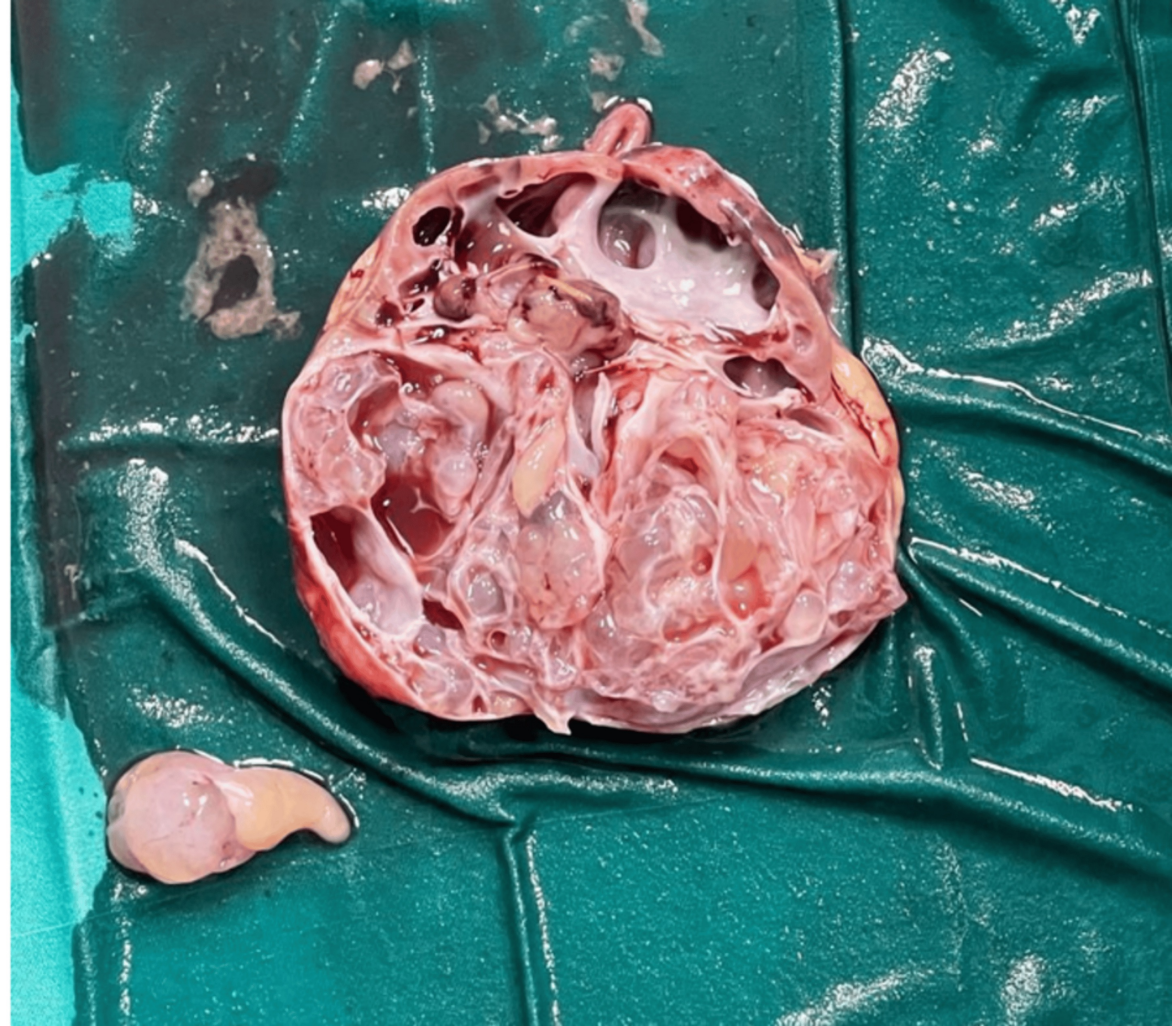
Gross specimen of the excised left renal cystic mass showing a well-encapsulated, multiloculated lesion with multiple clear fluid-filled cysts and thin fibrous septa, consistent with cystic nephroma.

On gross pathology, the kidney specimen (8.2 × 7.2 × 4.8 cm) was encapsulated and bosselated. The cut surface showed numerous variably sized (0.2-2 cm) clear-fluid cysts, separated by thin fibrous septa, involving all poles of the kidney (Figure 4). There was no solid component or focal necrosis visible, and the cysts did not communicate with one another or with the renal pelvis. A rim of normal renal parenchyma was preserved at the periphery.

Histopathology

Microscopic examination revealed features classic for CN. Each cyst was lined by a single layer of flattened to low cuboidal epithelial cells, with focal hobnail change. The intervening septa were fibrous and contained only mature elements (some tubules) and mild chronic inflammation - importantly, no primitive blastemal, epithelial, or stromal (Wilms-type) elements were identified in any septum. The remaining renal cortex showed changes of chronic obstruction (hyaline casts, thyroidization), consistent with hydronephrosis. All sampled lymph nodes (infra-hilar, para-aortic, mesenteric) showed only reactive hyperplasia and no tumor. The final diagnosis was multiloculated CN with negative margins.

Follow-up ultrasonography performed postoperatively revealed no evidence of residual or recurrent lesion.

## Discussion

The imaging findings in this case aligned closely with the underlying pathology. The CT and ultrasound appearance of a multiloculated cystic mass with only thin enhancing septa corresponds directly to the histologic structure of CN: multiple clear-fluid cysts separated by fibrous connective septa [1,2]. On CT, the enhancing septa (and capsule) reflect the fibrous tissue visualized microscopically [3,4], whereas the non-enhancing fluid-filled cysts correspond to the dilated epithelial-lined spaces. The absence of any enhancing solid component on imaging correlates with the lack of blastemal or solid elements on pathology [2]. The gross specimen’s smoothly encapsulated, multilocular architecture is mirrored by the well-demarcated margins on imaging. Notably, the CT finding of locules herniating into the dilated collecting system reflects a common gross feature of CN: large cysts may bulge into the renal pelvis without true communication. Thus, the radiologic appearance (simple cysts with thin septa) was entirely concordant with the benign histologic diagnosis.

Multilocular CN is a benign pediatric renal tumor that poses a diagnostic challenge because of its overlap with other cystic lesions. Although most pediatric patients present with a painless, enlarging abdominal mass, some cases may be detected incidentally during imaging performed for unrelated indications or may present with mild, nonspecific symptoms. Awareness of this variability can aid early recognition and timely management [1]. Awareness of its imaging and pathologic signature is key to guiding management. In imaging, CN typically appears as a well-circumscribed, encapsulated, multicystic renal mass with only thin, uniform septa [2]. This mirrors descriptions from the literature: for example, Chung et al. [2] note that CN and CPDN are “grossly identical and cannot be distinguished at imaging,” both appearing as encapsulated, multiloculated cystic masses lacking nodular solid components. On ultrasound, CN’s cysts are usually anechoic (or have low-level echoes), with thin echogenic septa and minimal septal vascularity. On CT, the cyst fluid has water-like attenuation and the septa (and capsule) enhance; crucially, there are no discrete enhancing nodules or irregular soft-tissue components.

Differential diagnosis

The primary differentials for a pediatric multiloculated cystic kidney mass include:

Cystic Partially Differentiated Nephroblastoma (CPDN)

Radiographically, CPDN is indistinguishable from CN. Both present as entirely cystic masses with septa. Only histologic examination can separate them. In CPDN, the septa contain immature nephrogenic (blastemal) elements, whereas CN septa contain only mature stroma [1]. In our case, extensive sampling showed no immature or blastemal foci, effectively excluding CPDN.

Wilms Tumor (Cystic Variant)

Wilms tumor often has solid components. Imaging clues favoring Wilms include irregular, thickened septa, enhancing nodular tissue, hemorrhage, or calcification within a cystic mass. Wilms is also more likely in older children and may demonstrate local invasion or metastases. In contrast, CN lacks solid nodules and typically has a smooth contour [1,2]. In our patient, the absence of any enhancing solid nodule or invasive feature on CT argued against Wilms tumor (a “cystic nephroblastoma”), which correlates with the benign pathology.

Multicystic Dysplastic Kidney (MCDK)

MCDK is a developmental, non-neoplastic condition in which a kidney is replaced by variable cysts with dysplastic tissue. Imaging usually shows no normal renal tissue (no functioning parenchyma), and diagnosis is often made antenatally or at birth. Unlike CN, MCDK lacks a functioning collecting system (there is no single renal pelvis) and typically does not present as a discrete, encapsulated mass in an older child. In contrast, CN presents later, with preserved but compressed renal parenchyma along the periphery, as seen in this case.

In infants, congenital mesoblastic nephroma can present with cystic degeneration but is predominantly a solid spindle-cell tumor on imaging. Renal cell carcinoma (pediatric subtype) is extremely rare in this age group and usually appears as a solid mass. Complex renal cysts of other etiologies (e.g., hydatid cyst) are rare. The uniform imaging features here (multiloculated simple cysts) and benign histology effectively exclude these (Table 1).

**Table 1:** Imaging features of pediatric cystic nephroma and its differential diagnoses.

Feature	Cystic nephroma (CN)	Cystic partially differentiated nephroblastoma (CPDN)	Cystic Wilms tumor	Multicystic dysplastic kidney (MCDK)
Typical age	Infants/toddlers (<4 years)	Infants/young children	Usually 2-5 years	Prenatal/neonatal
Nature	Benign multiloculated cystic neoplasm	Low malignant potential	Malignant renal tumor	Developmental anomaly
Margins	Well-circumscribed, encapsulated	Well-circumscribed, encapsulated	May be lobulated/infiltrative	Irregular dysplastic kidney
Internal architecture	Multiloculated cysts with thin septa	Multiloculated cysts with septa	Mixed solid-cystic/necrotic	Multiple noncommunicating cysts
Solid enhancing nodules	Absent	Usually absent radiologically	Often present	Absent
Septal enhancement	Thin, uniform septal enhancement	Thin/mildly thick septa	Thick irregular septa	Minimal/absent
Calcification	Rare	Rare	May be present	Rare
Collecting system involvement	May show herniation into pelvis/calyces	Possible compression	Distortion/invasion possible	Poorly developed/absent collecting system
Renal parenchyma	Peripheral compressed preserved parenchyma	Compressed residual parenchyma	Variable destruction	Dysplastic/absent normal parenchyma
Lymphadenopathy/metastasis	Absent	Usually absent	May be present	Absent
Histopathology	Fibrous septa with mature stroma only	Septa contain blastemal/nephrogenic rests	Blastemal/stromal/epithelial malignant elements	Dysplastic tissue, cartilage, primitive ducts
Prognosis	Excellent after surgery	Good after excision	Depends on stage	Usually involutes/monitored

Management

Complete surgical excision is curative for CN. Given its benign nature, nephron-sparing resection can be considered if the tumor location and size permit. For instance, some reports advocate partial nephrectomy in CN to preserve renal tissue [5]. However, because preoperative imaging cannot guarantee benignity, many cases (including ours) undergo total nephrectomy. In this patient, the tumor’s large size and hydronephrosis precluded a partial approach, so a simple nephrectomy was performed with curative intent. No adjuvant therapy is indicated for CN. Follow-up imaging is advised chiefly to monitor the contralateral kidney; unlike CPDN (which can rarely recur), metastatic spread has not been reported for CN.

Genetic considerations

Although beyond the scope of routine radiology workup, it is worth noting that many pediatric CNs harbor DICER1 mutations as part of a familial tumor predisposition syndrome [6]. This association links CN with pleuropulmonary blastoma, ovarian Sertoli-Leydig tumors, and other rare neoplasms. In our patient, germline testing was not performed, but awareness of this link is important for counseling and surveillance in some clinical contexts.

## Conclusions

Pediatric CN should be included in the differential diagnosis for any young child with a well-defined, multiloculated renal cystic mass. Key radiologic clues are purely cystic architecture with thin enhancing septa and absence of solid nodules. Nonetheless, imaging cannot distinguish CN from its malignant mimics, so histopathologic confirmation is required. Radiologists should recognize CN’s characteristic imaging features and coordinate with the clinical and pathology teams to avoid overtreatment. When identified, the prognosis is excellent, with surgical resection alone allowing nephron-sparing approaches whenever feasible. 
